# Implementação do Programa Boas Práticas em Cardiologia adaptado do *Get With The Guidelines* ^*®*^ em Hospitais Brasileiros: Desenho do Estudo e Fundamento

**DOI:** 10.36660/abc.20190393

**Published:** 2020-07-28

**Authors:** Fabio Papa Taniguchi, Sabrina Bernardez-Pereira, Suzana Alves Silva, Antônio Luiz Pinho Ribeiro, Louise Morgan, Anne B. Curtis, Kathryn Taubert, Denilson Campos de Albuquerque, Bernadete Weber, Pedro Paulo Magalhães Chrispim, Camila Pereira Pinto Toth, Erica Deji Moura Morosov, Gregg C. Fonarow, Sidney C Smith, Angelo Amato Vincenzo de Paola

**Affiliations:** 1 Hospital do Coração São PauloSP Brasil Hospital do Coração, São Paulo, SP – Brasil; 2 Universidade Federal de Minas Gerais Belo HorizonteMG Brasil Universidade Federal de Minas Gerais (UFMG), Belo Horizonte, MG – Brasil; 3 American Heart Association Inc DallasTexas EUA American Heart Association Inc, Dallas, Texas – EUA; 4 University at Buffalo BuffaloNew York EUA University at Buffalo -The State University of New York, Buffalo, New York – EUA; 5 American Heart Association Switzerland American Heart Association Switzerland Basel Suíça American Heart Association Switzerland, Basel – Suíça; 6 Universidade do Estado do Rio de Janeiro Rio de JaneiroRJ Brasil Universidade do Estado do Rio de Janeiro (UERJ), Rio de Janeiro, RJ – Brasil; 7 University of California Los Angeles Los AngelesCalifórnia EUA University of California Los Angeles, Los Angeles, Califórnia – EUA; 8 University of North Carolina at Chapel Hill Chapel HillNorth Carolina EUA University of North Carolina at Chapel Hill, Chapel Hill, North Carolina – EUA; 9 Universidade Federal de São Paulo Escola Paulista de Medicina São PauloSP Brasil Universidade Federal de São Paulo Escola Paulista de Medicina, São Paulo, SP – Brasil

**Keywords:** Doenças Cardiovasculares/fisiopatologia, Insuficiência Cardíaca, Fibrilação Atrial, Síndrome Coronariana Aguda, Melhoria de qualidade/tendências, Guias como Assunto

## Abstract

**Fundamento:**

Existem grandes oportunidades de melhoria da qualidade do cuidado cardiovascular em países em desenvolvimento por meio da implementação de um programa de qualidade.

**Objetivo:**

Avaliar o efeito de um programa de Boas Práticas em Cardiologia (BPC) nos indicadores de desempenho e desfechos clínicos dos pacientes relacionados à insuficiência cardíaca, fibrilação atrial e síndromes coronarianas agudas em um subconjunto de hospitais públicos brasileiros.

**Métodos:**

O programa Boas Práticas em Cardiologia (BPC) foi adaptado do programa *Get With The Guidelines* (GWTG) da *American Heart Association* (AHA) para ser utilizado no Brasil. O programa está sendo iniciado em três domínios de cuidado simultaneamente (síndrome coronariana aguda, fibrilação atrial e insuficiência cardíaca), o que consiste em uma abordagem nunca testada no GWTG. Existem seis eixos de intervenções utilizadas pela literatura sobre tradução do conhecimento que abordará barreiras locais identificadas por meio de entrevistas estruturadas e reuniões regulares para auditoria e feedback. Planeja-se incluir no mínimo 10 hospitais e 1500 pacientes por doença cardíaca. O desfecho primário inclui as taxas de adesão às medidas de cuidado recomendadas pelas diretrizes. Desfechos secundários incluem o efeito do programa sobre o tempo de internação, mortalidade global e específica, taxas de readmissão, qualidade de vida, percepção do paciente sobre saúde e adesão dos pacientes às intervenções prescritas.

**Resultados:**

Espera-se, nos hospitais participantes, uma melhoria e a manutenção das taxas de adesão as recomendações baseadas em evidência e dos desfechos dos pacientes. Este é o primeiro programa em melhoria da qualidade a ser realizado na América do Sul, que fornecerá informações importantes de como programas de sucesso originados em países desenvolvidos como os Estados Unidos podem ser adaptados às necessidades de países com economias em desenvolvimento como o Brasil. Um programa bem sucedido dará informações valiosas para o desenvolvimento de programas de melhoria da qualidade em outros países em desenvolvimento.

**Conclusões:**

Este estudo de mundo real proverá informações para a avaliação e aumento da adesão às diretrizes de cardiologia no Brasil, bem como a melhora dos processos assistenciais. (Arq Bras Cardiol. 2020; 115(1):92-99)

## Introdução

O sistema de saúde público no Brasil atende cerca de 70% da população do país e atua como o serviço de atenção primária à saúde.^1^ Apesar de inúmeras iniciativas do governo federal para melhorar a eficiência do sistema de saúde brasileiro, seus resultados têm sido inconsistentes, indicando uma grande necessidade de melhoria.^1 , 2^ Além disso, pouco tem sido feito para se controlar a subutilização ou o uso excessivo de recursos destinados à saúde, e as barreiras para a adoção de terapias baseadas em evidências em todo o país.^2^

Tem se observado grande variabilidade na qualidade do cuidado, avaliada por instituições de saúde no Brasil por meio de medidas de desempenho, com o apoio da Sociedade Brasileira de Cardiologia (SBC).^3 - 5^ Iniciativas educativas e programas de melhoria da qualidade (MQ) têm ajudado a melhorar o cuidado oferecido a pacientes com doença cardiovascular (DCV).^6 , 7^ Assim, intervenções clínicas bem alinhadas, tal como o programa de MQ plurianual da *American Heart Association* (AHA) intitulado *Get With the Guidelines* (GWTG), se adaptadas às diretrizes e ao sistema de saúde do Brasil, poderiam ter impactos significativos sobre o tratamento e desfechos de pacientes com DCV e padrões de conduta por parte dos cuidadores.

O GWTG é um programa de MQ criado pela AHA e a Associação Americana de Acidente Vascular Cerebral (ASA, *American Stroke Association* ) com o objetivo de melhorar o cuidado de pacientes com DCV hospitalizados. O programa foi criado para auxiliar hospitais em reformular o cuidado a doenças cardíacas de alto custo econômico, tais como síndrome coronariana aguda (SCA), fibrilação atrial (FA), insuficiência cardíaca (IC) e acidente vascular cerebral, e validado nos Estados Unidos nos últimos 17 anos. O programa melhorou a qualidade do cuidado oferecido durante a internação hospitalar, bem como desfechos dos pacientes e custos.^8^

É dentro desse contexto, após adaptação apropriada ao sistema de saúde brasileiro, que este novo programa está sendo lançado. Seu principal objetivo é avaliar a taxa de adesão de profissionais da saúde que trabalham em hospitais às recomendações mais recentes das diretrizes da AHA e da SBC sobre IC, FA e SCA, bem como seu efeito sobre os desfechos e a qualidade de vida dos pacientes antes e após a implementação do programa Boas Práticas em Cardiologia (BPC), adaptado da iniciativa GWTG. Tal iniciativa no Brasil é resultado de uma colaboração tripartite entre a AHA, a SBC e o Ministério da Saúde, com participação do Hospital do Coração (HCor), para ser testada em alguns hospitais públicos e, se comprovada sua eficiência, para ser implementada em todo o país.

## Métodos

O BPC é um programa de MQ que foi adaptado do GWTG e aprovado pelo Comitê de Ética do centro coordenador (sob o número 48561715.5.1001.0060). O programa será implementado em hospitais terciários das cinco macrorregiões do Brasil. Os comitês e grupos de gerenciamento do estudo estão descritos no Apêndice 1.

Após aceitação em participar do estudo pelas instituições e aprovação dos comitês de ética locais, o grupo gestor fará uma visita inicial para assegurar que a instituição possui a infraestrutura necessária para participar do programa, e para apresentar o programa à direção da instituição.

O efeito do programa sobre o desempenho da instituição, a qualidade de vida e desfechos clínicos será avaliado em um estudo coorte quase-experimental por meio de coleta de dados antes e após a implementação do programa BPC.

Antes da intervenção, a avaliação será realizada durante um período de aproximadamente dois meses antes da implementação do programa BPC na instituição, ou após a inclusão dos primeiros 15 pacientes em cada braço. A avaliação pós-intervenção será realizada após a primeira intervenção, e terá duração de aproximadamente 18 meses. Os pacientes serão acompanhados por telefone um mês e seis meses após alta hospitalar por entrevistadores treinados locais.

Uma equipe multidisciplinar composta de um líder local, médicos, enfermeiros, e educadores será responsável por estabelecer estratégias locais para melhoria, e reunir esforços para o programa local.

### População

Serão considerados elegíveis pacientes com idade igual ou superior a 18 anos, admitidos nos hospitais selecionados com um diagnóstico primário de IC aguda (CID-10 I50; I50.0; I50.1 ou I50.9), SCA (CID-10 códigos I20.0 a I21.9 e I22.0 a I22.9) ou FA/flutter atrial (CID-10 I-48), independentemente de história prévia de quaisquer dessas condições, e que aceitem a participar do estudo mediante assinatura do termo de consentimento. A identificação de pacientes com FA ou flutter atrial será realizada no ambulatório. Os critérios de elegibilidade estão descritos em detalhes no Apêndice 2.

### Definição das medidas de desempenho e métricas de qualidade

As medidas de desempenho e métricas de qualidade foram selecionadas daquelas sugeridas pelo Colégio Americano de Cardiologia (ACC, *American College of Cardiology* )/AHA para IC,^9^ SCA^10^ e FA^11^ para compor dois conjuntos de indicadores para cada uma dessas condições. Como descrito anteriormente, o primeiro conjunto de indicadores derivaram das recomendações classe I das últimas diretrizes do ACC/AHA e incluíram comentários públicos e um processo de revisão por pares, enquanto o segundo conjunto derivou-se de outras recomendações sem seguir uma metodologia restrita.^12 , 13^ Essas métricas de desempenho e de qualidade foram então revisadas e adaptadas às diretrizes atuais no Brasil.

Foram selecionadas 21 medidas de desempenho, cinco para IC, nove para SCA e sete para FA ( Tabela 1 ). Outras 22 métricas de qualidade foram incluídas nos três braços do programa, nove para IC, seis para SCA e sete para FA (Apêndice 3). Pacientes elegíveis são definidos como aqueles sem intolerância ou contraindicações documentadas às medidas específicas.

**Tabela 1:** – Medidas de desempenho

Tempo	Medida de desempenho	Definição	IC	FA	SCA
Dentro de 24h da admissão	Aspirina precoce*	Proporção de pacientes com SCA que recebem aspirina nas 24 horas a partir da chegada ao hospital			●
	Terapia de reperfusão adequada	Proporção de pacientes com IAMCST submetidos a trombólise nos primeiros 30 minutos ou angioplastia primária dentro de 90 minutos da chegada ao hospital			●
Durante hospitalização	Avaliação dos fatores de risco para tromboembolismo	Proporção de pacientes com FA não valvar/flutter atrial com uma avaliação pelo escore de risco CHADS2-VASc documentada		●	
	Avaliação do risco de sangramento	Proporção de pacientes com avaliação de risco por HAS-BLED documentada		●	
	Avaliação da função ventricular esquerda	Proporção de pacientes com IC com algum registro da função do ventrículo esquerdo, seja no prontuário médico ou relatórios outros relatórios acessíveis nos registros hospitalares 12 meses antes da admissão ou durante a hospitalização, ou com uma avaliação médica agendada após a alta	●		
Na alta	Aspirina*	Proporção de pacientes com SCA com prescrição de aspirina na alta			●
	IECA/BRA*	Proporção de pacientes com IC com FEVE < 40% ou pacientes com SCA com FEVE < 45% com prescrição de IECA/BRA na alta	●	●	●
	Betabloqueadores*	A proporção de pacientes com IC com FEVE ≤ 40% em uso de um betabloqueador com eficácia comprovada (Bisoprolol, Carvedilol, Succinato de Metoprolol CR/XL) prescrito na alta	●	●	●
		Proporção de pacientes com SCA com um betabloqueador prescrito na alta			
		Proporção de pacientes com FA com FEVE ≤ 40% ou DAC com prescrição de betabloqueador na alta			
	Anticoagulantes*	Proporção de pacientes com FA e alto risco de tromboembolismo de acordo com o escore CHADS2_VASc, em uso de anticoagulantes		●	
	Estatinas*	Proporção de pacientes com FA e DAC, AVC/AIT, DVP ou diabetes, com prescrição de estatina na alta		●	●
		Proporção de pacientes com SCA sem contraindicações, com prescrição de estatina para controle de LDL na alta			
	Inibidores de aldosterona*	Proporção de pacientes com IC e FEVE ≤ 35% em uso de inibidores de aldosterona	●		
	Controle da pressão sanguínea	Proporção de pacientes com SCA em uso de medicamentos para controle da pressão sanguínea			●
	Aconselhamento para parar de fumar	Proporção de pacientes com SCA, fumantes ativos nos últimos 12 meses, que receberam aconselhamento para parar de fumar durante a internação ou na alta			●
	Visita de retorno	Proporção de pacientes com FA que receberam alta usando Varfarina e tiveram um planejamento de retorno para acompanhamento do INR antes da alta		●	
	Consulta pós-alta	Proporção de pacientes com IC com visita de acompanhamento agendada e documentada	●		

* Somente pacientes elegíveis, sem contraindicações, serão computados no denominador. SCA: síndrome coronária aguda, IECA: inibidor de enzima conversora de angiotensina, FA: fibrilação atrial, BRA: bloqueador de receptor de angiotensina; DAC: doença arterial coronariana; AVC: acidente vascular cerebral; IC: insuficiência cardíaca; RNI: razão normalizada internacional; LDL: lipoproteína de baixa densidade; FEVE: fração de ejeção do ventrículo esquerdo; IAMCST: infarto agudo do miocárdio com elevação do segmento; DVP: doença vascular periférica; AIT: acidente isquêmico transitório.

As taxas de adesão às recomendações serão medidas utilizando-se abordagem baseada em oportunidades segundo metodologia recomendada pelo ACC e AHA.^14^

### Medidas de desfecho

Tempo de internação, mortalidade hospitalar, mortalidade por doença cardíaca um mês e seis meses, e ocorrência de reinternação durante um mês e seis meses por uma causa relacionada ao índice de admissão serão computados.

Além disso, serão medidas qualidade de vida pelo questionário WHOQOL-BREF^15^ e a percepção em saúde pelo Numering Rating Scale (NRS)^16^ na alta hospitalar e seis meses depois.

### Identificação de barreiras no tempo basal

Possíveis causas de não adesão às diretrizes que requeiram intervenções específicas serão identificadas por discussão com as instituições, por meio de entrevista semiestruturada (Apêndice 4). A entrevista semiestruturada será realizada antes do início do projeto para mapear processos institucionais e fluxo do cuidado em cada braço no qual a instituição participa. Essas entrevistas têm o objetivo de identificar mudanças comportamentais específicas necessárias para estimular a participação no programa BPC e a adesão às recomendações das diretrizes. Assim, quando os processos do cuidado falham em implementar as terapias recomendadas, mudanças podem ser implementadas para melhorar um processo ou cuidado específico.

### Coleta de dados

Dados clínicos dos pacientes incluídos serão registrados em uma base de dados em rede (MySQL versão 5.7 ou mais recente) desenvolvida especificamente para este projeto. Cada instituição será responsável pela coleta de seus dados, que será realizada por uma equipe treinada, sob a supervisão do líder local. Os dados serão extraídos dos prontuários médicos e entrevistas estruturadas realizadas diretamente com os pacientes durante a hospitalização após um mês e seis meses de acompanhamento.

Os dados incluirão dados demográficos, comorbidades e fatores de risco, sintomas na admissão, alfabetização em saúde, perfil de risco de acordo com padrões internacionais para cada braço do programa,^17 - 21^ tratamentos e procedimentos realizados dentro e fora do hospital, medicamentos na alta hospitalar e prevenção secundária, aconselhamento na alta e adesão dos pacientes às recomendações.

### Manejo dos dados e controle de qualidade

Todos os dados serão tratados como informação protegida e armazenados em segurança em um servidor de rede, protegido por senha, e acessível em tempo real por meio de um navegador da web, por qualquer usuário aprovado.

A acurácia e a integridade dos dados serão asseguradas seguindo-se as mesmas metodologias do GWTG.^22 , 23^

### Intervenções de MQ e reconhecimento do hospital

**Figura 1:**
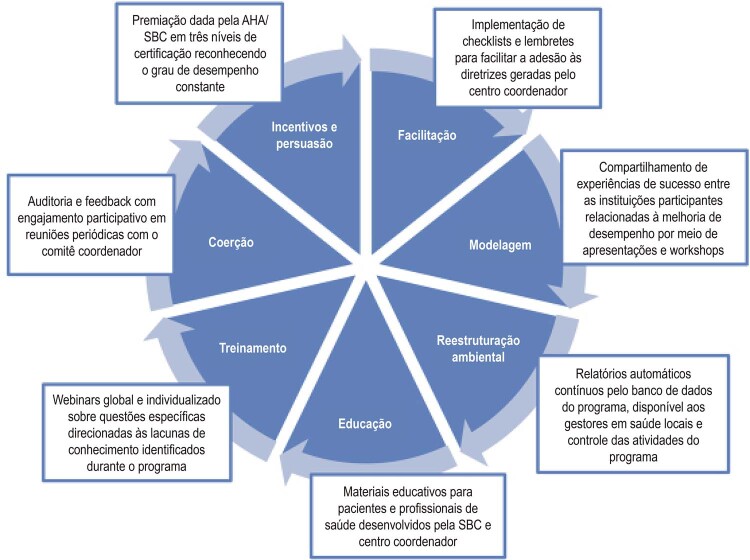
– Eixos de intervenção *Meta de mudança de comportamento: profissionais de saúde &Meta de mudança de comportamento: Pacientes e profissionais da saúde # Meta de mudança de comportamento: gestores de saúde.

Diferentemente da abordagem feita nos EUA, o programa brasileiro utiliza uma estrutura didática baseada no estudo de Michie et al.,^24^ As intervenções foram agrupadas em sete domínios com objetivo de provocar mudanças comportamentais (facilitação e restrição; modelagem; reestruturação do meio ambiente; educação; incentivos e persuasão; coerção; e treinamento). Esses grupos de intervenção serão implementados em todas as instituições participantes e poderão ser enfatizados individualmente no decorrer do estudo de acordo com as barreiras identificadas no tempo basal e com os relatórios mensais sobre adesão total e específica às recomendações. A descrição das intervenções em cada um desses grupos encontra-se na Figura 1 .

A coordenação dessas intervenções será realizada por um enfermeiro membro do grupo gestor, e será feita por meio de checklists e lembretes, webinars, relatórios automáticos e relatórios em tempo real por um banco de dados eletrônico, materiais educativos, reuniões quinzenais para auditoria e feedback, e reconhecimentos e treinamento dos hospitais quanto a metodologias de MQ para implementação de ciclos rápidos de melhoria usando instrumentos recomendados pelo IHI, *Institute for Healthcare Improvement* .^25 , 26^ Serão usados no decorrer do estudo, conceitos de melhoria tais como treinamento de equipe de MQ e estabelecimento de metas com base nas barreiras que necessitam ser superadas, e monitoramento e análise dos resultados.

Os relatórios eletrônicos captarão informações em tempo real ao serem preenchidos na base de dados eletrônica do estudo. Nos relatórios, serão incluídos gráficos de execução específicos descrevendo as tendências temporais mensais das taxas de adesão global e específica da instituição em relação a uma meta pré-estabelecida de 85% e às taxas medianas observadas no período selecionado para aquela mesma instituição.^27^ Cada instituição poderá ver, em tempo real, seus próprios gráficos, bem como os gráficos mostrando as taxas médias das outras instituições participantes (em anonimato). O centro coordenador poderá acompanhar todas as instituições participantes concomitantemente.

Para este projeto, estabelecemos como meta um limiar de 85% com base em resultados prévios do GWTG que mostraram uma melhora nos desfechos clínicos quando as instituições alcançaram esse limiar.^28^ Os hospitais receberão um prêmio em bronze pela SBC caso alcancem esse limiar por pelo menos três meses consecutivos, um prêmio em prata caso mantenham esses resultados por pelo menos seis meses, e um prêmio de ouro caso continuem no limiar (ou acima) por 12 meses consecutivos.

### Análise dos dados

A análise dos dados será realizada utilizando-se o programa R versão 3.4.0 ou mais recente.

Os hospitais serão excluídos da análise de determinada medida de desempenho se no denominador houver um número menor que 10 pacientes.

As variáveis contínuas com distribuição normal serão apresentadas em média e desvio padrão, e aqueles com distribuição não normal como mediana e percentis (25^o^ e 75^o^). As variáveis ordinais ou categóricas serão apresentadas como frequências absolutas, porcentagens e intervalos de confiança de 95%. Dados faltantes serão abordados de acordo com cada análise e considerados como não adesão para a medida específica.

O efeito longitudinal do programa sobre IC, SCA e FA será avaliado comparando-se as taxas globais de adesão às recomendações antes e após sua implementação nas instituições participantes quinzenalmente, utilizando-se um modelo linear generalizado misto (MLGM) para análise de tendência temporal por um horizonte de tempo de 18 meses. O efeito será expresso em médias de proporções e seus respectivos intervalos de confiança de 95%. Espera-se que a abordagem de efeitos aleatórios do MLGM detecte diferenças basais entre as instituições.^29^

Escores de qualidade de vida serão calculados utilizando-se a metodologia descrita no manual do questionário WHOQOL-BREF.^30^ O escore total consiste na média dos escores dos quatro domínios do instrumento (saúde física, saúde psicológica, relações sociais e meio ambiente).^30^ A consistência interna do instrumento será calculada utilizando-se o coeficiente alfa de Cronbach. Um valor acima de 0,7 será considerado apropriado.

Os resultados das variáveis dependentes de mortalidade, taxa de readmissão, tempo de internação, variação na qualidade de vida e na percepção em saúde, observados ao longo do tempo nas instituições participantes, serão ajustados pelo MLGM mutivariado quanto às variáveis demográficas, clínicas e socioeconômicas, gravidade da doença, fatores de risco, percepção da saúde (NRS), nível de alfabetismo em saúde, e grau de adesão geral e específica da instituição às recomendações clínicas. As variáveis serão incluídas no modelo quando estiverem associadas na análise univariada e bivariada (p < 0,10) e de acordo com a relevância clínica. Serão calculados odds ratio e riscos relativos, conforme apropriado, com respectivo IC95%.

Todas as análises serão bicaudais e realizadas de maneira independente em cada braço do protocolo, adotando-se um nível de significância de 0.05.

## Discussão

### Por que este projeto é necessário?

No Brasil, um país vasto e com um sistema universal de saúde complexo,^1^ a qualidade do cuidado cardiovascular tem sido o tema de avaliação e preocupação. O acesso dos pacientes aos vários níveis de saúde varia por todo o país e a qualidade do cuidado oferecido é bastante heterogêneo.^1 , 2^

Assim como em outras partes do mundo e apesar de esforços da sociedade médica em publicar diretrizes clínicas, a mortalidade relacionada às DCVs ainda é alta, refletindo a dificuldade dos pacientes em terem acesso a terapias e cuidados recomendados em tempo apropriado.^31 , 32^

Registros realizados pela SBC em várias regiões do Brasil mostraram uma alta variação na qualidade do cuidado a condições cardiovasculares de alto encargo econômico,^32 , 33^ tais como doença arterial coronariana (DAC),^3 , 34^ IC,^4^ acidente vascular cerebral, e FA.^35^ Esses registros mostraram que a adesão a terapias baseadas em evidência ainda é insuficiente e, pelo menos para IC, a ausência de terapias adequadas é mais crítica em instituições públicas não acadêmicas das regiões mais pobres do Brasil.^4^ Também se observou que a morbidade e a mortalidade relacionadas à IC são muito maiores que àquelas em países desenvolvidos, mesmo após ajuste por região, número de leitos hospitalares e tipo de instituição. Os registros brasileiros deram uma grande contribuição ao demonstrar como tem sido a abordagem dessas condições altamente prevalentes em todo o país. No entanto, não têm abordado a lacuna existente na implementação dessas intervenções que têm dificultado a ocorrência de melhorias na qualidade do cuidado. Ainda, esses registros não controlaram situações em que terapias específicas não são recomendadas ou são contraindicadas.^3 , 4 , 34 , 35^

Os dois ensaios randomizados realizados no Brasil (BRIDGE-ACS e IMPACT-AF) para testar intervenções multifacetadas para melhorar a adesão às recomendações de diretrizes mostraram que a implementação de intervenções de MQ é viável e pode ser eficaz.^6 , 7^ Contudo, esses estudos não levaram em consideração barreiras relacionadas ao contexto local, não testaram se os resultados observados sobre adesão às recomendações são mantidos ao longo do tempo, ou o efeito das intervenções na qualidade de vida dos pacientes.^6 , 7^ O estudo BRIDGE-ACS, por exemplo, que foi realizado principalmente em instituições acadêmicas,^36^ atingiu no máximo 68% de adesão a terapias agudas e somente 51% se fossem consideradas todas as terapias agudas e na alta, sem impacto na mortalidade em 30 dias.^6^ O programa GWTG mostra que os hospitais que atingem 85% de adesão a terapias baseadas em evidência alcançaram melhores resultados em desfechos clínicos.^37 , 38^

Esses achados fornecem um argumento convincente para a implementação da iniciativa de MQ em hospitais brasileiros que considera a complexidade da realidade local e que já foi testada e cuja eficácia demonstrada previamente. O programa GWTG, implementado em cerca de 50% dos hospitais nos EUA, mostrou um efeito duradouro sobre a mortalidade, tempo de internação e custos.^39^ Existe, então, um potencial de diminuição do encargo econômico imposto pela SCA, IC, e FA sobre o sistema de saúde brasileiro.

### Em que se difere o programa brasileiro?

O programa GTWG funciona nos EUA há mais de 15 anos. Apesar disso, somente recentemente, em 2016, um outro país (a China) beneficiou-se de um programa similar para SCA.^36^ No Brasil, estamos iniciando o programa em três dimensões – SCA, FA e IC. Um programa de qualidade de abrangência nacional, com foco em diferentes condições, que inclua ambulatórios ainda não foi testado no programa GWTG.^8 , 22^ Ainda, a ideia de desfechos relatados pelo próprio paciente, incluindo a qualidade de vida, foi contemplada pelo programa BPC e pode ajudar ministérios e sociedades de cardiologia em direcionar políticas de saúde às necessidades locais.

A identificação de barreiras e facilitadores em cada hospital é considerada uma das etapas chaves no sucesso de estratégias de implementação clínica. Neste projeto, estamos utilizando, como modelo conceitual, uma estrutura didática proposta por Michie, Stralen e West,^24^ que integra uma ampla gama de mecanismos dinâmicos e interativos para promover mudanças comportamentais resultantes da interação entre o indivíduo (capacidade e motivação) e o meio ambiente (oportunidades).^24^ Esse modelo também auxiliará o centro coordenador em identificar e agir sobre necessidades institucionais específicas durante o projeto. Dessa maneira, em algumas instituições, o foco da intervenção será melhorar a capacidade, enquanto em outras será aumentar a motivação, ou ainda, aumentar ou restringir o fornecimento de oportunidades, individualmente ou em conjunto, dependendo dos objetivos de cada instituição. Intervenções como o programa de premiações que foi considerado uma das chaves para o sucesso no programa GWTG será enfatizado em todas as instituições participantes.^40^

Aprendizados da experiência *escola aberta* do IHI, que incluiu a formatação da intervenção de auditoria e feedback por meio de gráficos de execução, também serão usados neste projeto.^41^ Essas abordagens consideram dados institucionais longitudinais de métricas de qualidade, não só em relação aos padrões médios de referência de outras instituições, como também aos objetivos estabelecidos para aquela instituição pela linha mediana de escores para todo o período de observação.^27 , 41^ Essa curva de feedback permite que a instituição se avalie continuamente e redefina processos em ciclos rápidos de melhoria,^25 , 26^ considerando como seu desempenho difere-se do objetivo e se ajustes feitos em suas intervenções multidisciplinares estão resultando em melhoria constante.

## Conclusão

Este novo programa de MQ será oferecido a instituições públicas brasileiras selecionadas e abordará questões referentes ao contexto local que permitirá a identificação de barreiras específicas à adoção de padrões de cuidado. Ele tem o potencial de prover soluções que possam resultar em melhoria constante na adesão a terapias baseadas em evidências e desfechos dos pacientes.

Espera-se que as estratégias implementadas contribuam para a criação de uma cultura organizacional focada na construção e troca de conhecimento entre instituições em todo o país, promovendo, assim, o avanço na qualidade do cuidado da cardiologia no Brasil.
