# Supplementary material for: Implementação do Programa Boas Práticas em Cardiologia adaptado do Get With The Guidelines ® em Hospitais Brasileiros: Desenho do Estudo e Fundamento
Source: Arq Bras Cardiol. 2020 Jul 28;115(1):92–9. [Article in Portuguese] doi: 10.36660/abc.20190393 (PMC8384317; doi:10.36660/abc.20190393)
Supplement: Translation

# Translation: Implementation of a Best Practice in Cardiology (BPC) Program Adapted from Get With The Guidelines^®^in Brazilian Public Hospitals: Study Design and Rationale

**Authors:** Fabio Papa Taniguchi, Sabrina Bernardez-Pereira, Suzana Alves Silva, Antônio Luiz Pinho Ribeiro, Louise Morgan, Anne B. Curtis, Kathryn Taubert, Denilson Campos de Albuquerque, Bernadete Weber, Pedro Paulo Magalhães Chrispim, Camila Pereira Pinto Toth, Erica Deji Moura Morosov, Gregg C. Fonarow, Sidney C Smith, Angelo Amato Vincenzo de Paola

**Affiliations:** 1 Brasil Hospital do Coração, São Paulo, SP – Brasil; 2 Brasil Universidade Federal de Minas Gerais (UFMG), Belo Horizonte, MG – Brasil; 3 EUA American Heart Association Inc, Dallas, Texas – EUA; 4 EUA University at Buffalo -The State University of New York, Buffalo, New York – EUA; 5 Suíça American Heart Association Switzerland, Basel – Suíça; 6 Brasil Universidade do Estado do Rio de Janeiro (UERJ), Rio de Janeiro, RJ – Brasil; 7 EUA University of California Los Angeles, Los Angeles, Califórnia – EUA; 8 EUA University of North Carolina at Chapel Hill, Chapel Hill, North Carolina – EUA; 9 Brasil Universidade Federal de São Paulo Escola Paulista de Medicina, São Paulo, SP – Brasil

**Keywords:** Cardiovascular Diseases/physiopathology, Heart Failure, Atrial Fibrillation, Acute Coronary Syndrome, Quality Improvement/trends, Guidelines as Topic

## Abstract

**Background:**

There are substantial opportunities to improve the quality of cardiovascular care in developing countries through the implementation of a quality program.

**Objective:**

To evaluate the effect of a Best Practice in Cardiology (BPC) program on performance measures and patient outcomes related to heart failure, atrial fibrillation and acute coronary syndromes in a subset of Brazilian public hospitals.

**Methods:**

The *Boas Práticas em Cardiologia* (BPC) program was adapted from the American Heart Association’s (AHA) Get With The Guidelines (GWTG) Program for use in Brazil. The program is being started simultaneously in three care domains (acute coronary syndrome, atrial fibrillation and heart failure), which is an approach that has never been tested within the GWTG. There are six axes of interventions borrowed from knowledge translation literature that will address local barriers identified through structured interviews and regular audit and feedback meetings. The intervention is planned to include at least 10 hospitals and 1,500 patients per heart condition. The primary endpoint includes the rates of overall adherence to care measures recommended by the guidelines. Secondary endpoints include the effect of the program on length of stay, overall and specific mortality, readmission rates, quality of life, patients’ health perception and patients’ adherence to prescribed interventions.

**Results:**

It is expected that participating hospitals will improve and sustain their overall adherence rates to evidence-based recommendations and patient outcomes. This is the first such cardiovascular quality improvement (QI) program in South America and will provide important information on how successful programs from developed countries like the United States can be adapted to meet the needs of countries with developing economies like Brazil. Also, a successful program will give valuable information for the development of QI programs in other developing countries.

**Conclusions:**

This real-world study provides information for assessing and increasing adherence to cardiology guidelines in Brazil, as well as improvements in care processes. (Arq Bras Cardiol. 2020; [online].ahead print, PP.0-0)

## Introduction

The Brazilian public health system serves about 70% of the country’s population and functions as Brazil’s primary health care delivery system.^1^ Despite a number of initiatives taken by the federal government to improve the efficiency of the Brazilian public health system, results have been inconsistent, indicating a great need for improvement.^1 , 2^ Furthermore, little has been done to control the under- or overutilization of healthcare resources and barriers that prevent evidence-based therapies from being implemented at the national level.^2^

Significant variability in the quality of care, assessed through performance measures by Brazilian health institutions with the support of the Brazilian Society of Cardiology (SBC), has been observed.^3 - 5^ Educational initiatives and programs for quality improvement (QI) have been shown to help improve care provided to patients with cardiovascular disease (CVD).^6 , 7^ Thus, a well-aligned clinical intervention such as a multiyear QI program like the American Heart Association (AHA) Get With The Guidelines (GWTG) program, if adapted to the guidelines and health care delivery system of Brazil, might have a significant impact on treatment and outcomes of CVD patients and practice patterns of their caregivers.

GWTG is a QI program created by the AHA and the American Stroke Association (ASA) with the aim of improving the care of patients hospitalized with CVD. It was created to assist hospitals in redesigning the care delivered for heart conditions of high economic burden such as acute coronary syndrome (ACS), atrial fibrillation (AF), heart failure (HF) and stroke and has been validated in the United States over the past 17 years. It has been shown to improve in-hospital quality of care, patient outcomes, and costs.^8^

It is within this context, after appropriate adaptation to the Brazilian healthcare system, that this novel program is being launched. Its main objective is to assess the adherence rates of hospital health professionals to the latest AHA/SBC guidelines’ recommendations on HF, AF and ACS and its effect on patient outcomes and quality of life before and after the implementation of a Best Practice in Cardiology (BPC) program adapted from the GWTG initiative. This initiative in Brazil is the result of a tripartite collaboration of the AHA, the SBC and the Brazilian Ministry of Health, with participation of the Hospital do Coração (HCor), to be tested in selected public hospitals and if proven effective, to be further implemented countrywide.

## Methods

BPC is a QI program that was adapted from GWTG and approved by the Institutional Review Board (IRB) of the Coordinating Center under the number 48561715.5.1001.0060. It will be implemented in selected tertiary hospitals of the Brazilian public health system in the five macro-regions of Brazil. The study steering committee and coordination groups are described in Appendix 1.

After acceptance to participate and local IRB approval, the project management group will make an initial visit to make sure that the center meets the infrastructure requirements to participate in the program and to present it to local leadership.

The effect of the program on measures of institutional performance, quality of life and clinical outcomes will be evaluated in a cohort quasi-experimental study design combined with a cohort design, through data collection before and after the implementation of the BPC Program.

Before the intervention, evaluation will occur over a period of approximately two months prior to the implementation of the BPC program in the institution or after the inclusion of the first 15 patients in each arm. Post-intervention evaluation will be conducted after the first intervention and will last approximately 18 months. Patients will be followed through telephone contact at one and six months after discharge by local trained interviewers.

A multidisciplinary team composed of a local leader, doctors, nurses, and patient educators will be responsible for establishing local strategies for improvement and driving the efforts to the local program.

### Population

Eligible patients will be consecutive patients aged 18 years or older, admitted to the selected hospitals with a primary diagnosis of acute HF (ICD-10 code I50; I50.0; I50.1 or I50.9), ACS (ICD10 codes: I20.0 to I21.9 and I22.0 to I22.9) or AF/Atrial Flutter (ICD-10 code I-48), regardless of a previous history of any of these conditions, and agree to participate in the study by signing an informed consent form. Screening for AF/flutter patients may be performed in the outpatient clinic. The details of eligibility criteria can be found in Appendix 2.

### Definition of performance measures and quality metrics

Performance measures and quality metrics were selected from the American College of Cardiology (ACC)/AHA care metrics on HF,^9^ ACS^10^ and AF^11^ to compose two sets of indicators for each of these conditions. As previously reported, the former set of indicators were derived from class I recommendations of the latest ACC/AHA guidelines and included public comment and a peer review process whereas the latter was derived from other recommendations not following a strict methodology.^12 , 13^ These performance and quality metrics have then been reviewed and adapted to be consistent with current guidelines in Brazil.

Twenty-one performance measures were selected, five for HF, nine for ACS and seven for AF ( Table 1 ). Twenty-two other quality metrics were included in the three arms of the program, nine for HF, six for ACS and seven for AF (Appendix 3). Eligible patients are defined as those patients without documented intolerance or contraindications for that specific measure.

**Table 1:** – Performance measures

Time	Performance measure	Definition	HF	AF	ACS
Within 24h of arrival	Early Aspirin*	Proportion of ACS patients receiving aspirin within 24 hours of hospital arrival			●
	Proper reperfusion therapy	Proportion of STEAMI patients submitted to thrombolysis within 30 min or primary angioplasty within 90 min from hospital arrival			●
During hospitalization	Assessment of thromboembolic risk factors	Proportion of non-valvular AF/Flutter patients with a documented CHADS2-VASc risk score assessment		●	
	Bleeding risk assessment	Proportion of patient with a documented HAS-BLED risk score assessment.		●	
	Assessment of left ventricle function	Proportion of HF patients with a documented LV function either in the medical records or other reports accessible in hospital charts in the 12 months before admission or during hospitalization or with a scheduled evaluation planned to be performed after discharge	●		
At discharge	Aspirin*	Proportion of ACS patients with aspirin prescribed at discharge			●
	ACEI/ARB*	Proportion of HF patients with LVEF < 40% or AF patients with LVEF ≤ 40% or ACS patients with LVEF < 45% with an ACEI/ARB prescribed at discharge	●	●	●
	Beta blockers*	Proportion of HF patients with LVEF ≤ 40% and a proven efficacious beta blocker (Bisoprolol, Carvedilol, Metoprolol Succinate CR/XL) prescribed at discharge	●	●	●
		Proportion of ACS patients with a beta blocker prescribed at discharge			
		Proportion or AF patients with either LVEF ≤ 40% or CAD with a beta blocker prescribed at discharge			
	Anticoagulants*	Proportion of AF patients at high risk for thromboembolism according to the CHADS2_VASc taking anticoagulants		●	
	Statin*	Proportion of AF patients with CAD, stroke/TIA, PVD or diabetes who were prescribed a statin at discharge		●	●
		Proportion of ACS patients without contraindications with statin prescribed for LDL control at discharge			
	Aldosterone inhibitors*	Proportion of HF patients with LVEF ≤ 35% taking aldosterone inhibitors	●		
	Blood pressure control	Proportion of ACS patients under medication for blood pressure control			●
	Smoke cessation counseling	Proportion of ACS patients, who are active smoker within the past 12 months, who receive smoking cessation advice during hospitalization or at discharge			●
	Returning visit appointment	Proportion of AF patients discharged on Warfarin who had an INR follow up planned prior to hospital discharge		●	
	Post-discharge appointment	Proportion of HF patients for whom a follow-up appointment was scheduled and documented	●		

* Only eligible patients, without contraindications, will be computed in the denominator. ACS: acute coronary syndrome; ACEI: angiotensin-converting enzyme inhibitor; AF: atrial fibrillation; ARB: angiotensin receptor blocker; CAD: coronary artery disease. CVA: cerebrovascular accident; HF: heart failure; INR: international normalized ratio; LDL: low density lipoprotein; LV: left ventricle; LVEF: left ventricle ejection fraction; PVD: peripheral vascular disease; STEAMI: ST elevation acute myocardial infarction; TIA: transient ischemic attack.

The overall rates of adherence to recommendations will be measured using an opportunity-based approach according to ACC/AHA methodology.^14^

### Outcome measures

Length of stay, in-hospital mortality, cardiac mortality at one month and at six months, and readmission within one month and six months due to a cause related to the index admission will be computed.

In addition, quality of life and health perception will be measured using the WHOQOL-BREF questionnaire^15^ and the Numering Rating Scale (NRS),^16^ respectively, at discharge and at six months.

### Identification of barriers at baseline

Possible causes of non-adherence to guidelines that require specific interventions will be identified through discussion with the institutions, via a semi-structured interview (Appendix 4). The semi-structured interview will be held before the start of the project for mapping institutional processes and flow of care in each arm in which the institution is enrolled. These interviews aim to identify specific behavioral changes needed to encourage participation in the BPC program as well as adherence to guideline recommendations. Thus, when care processes lead to failure to implement recommended therapies, changes can be implemented to improve a specific process or care.

### Data collection

Clinical data from the patients included will be registered on a web database (MySQL version 5.7 or higher) developed specifically for this project. Each hospital will be responsible for its own data collection by a trained local team of data abstractors who will work under the supervision of their local leadership. Data will be abstracted from medical charts and structured interviews made directly with the patients during hospitalization and at one and six months of follow-up.

Data will include demographics, comorbidities and risk factors, symptoms on arrival, health literacy, risk profile according to international standards for each arm of the program,^17 - 21^ in- and out-of-hospital treatment and procedures, discharge medications and secondary prevention, discharge counseling and patients’ adherence to recommendations.

### Data Management and Quality Control

All data will be treated as protected health information and securely stored centrally in a password-protected web server, accessible in real time by any approved user through a web browser.

Data accuracy and completeness will be ensured by following the same methodologies of the GWTG.^22 , 23^

### QI Interventions and Hospital Recognition

As opposed to the approach taken in the U.S., the Brazilian program uses a didactic framework based on Michie et al.^24^ Interventions were grouped in seven domains aiming to cause behavioral change (facilitation and restriction; modeling; environmental restructuring; education; incentives and persuasion; coercion; and training). These groups of interventions will be implemented in all participating institutions and can be emphasized individually throughout the study according to the barriers identified at baseline and to the monthly reports on overall and specific adherence to recommendations. The description of the interventions embedded in each of these groups is available in Figure 1 .

**Figure 1:** – Intervention axes *Target of behavior change: health professionals &Target of behavior change: Patients and health professionals # Target of behavior change: Health managers.

Coordination of these activities will be made by a nurse, member of the Management Group, and will include checklists and reminders, webinars, automatic and real time reports through an electronic database, educational materials, quarterly meetings for audit and feedback, and hospitals’ recognition and training on QI methodologies for the implementation of rapid improvement cycles by the use of the Institute for Healthcare Improvement (IHI) ’s tools.^25 , 26^ Concepts of improvement such as training of a QI team and establishment of goals based on the barriers that need to be overcame and monitoring and analysis of results will be used throughout the study.

The electronic reports will capture real time information when completed in the study’s electronic database. The reports will include specific run charts describing the temporal trends on a monthly basis of the overall and specific adherence rates of the institution in relation to an established goal of 85% and to the median rates observed in the selected period for that same institution.^27^ Each institution will be able to see, in real time, their own run charts and the charts showing average rates of the other participating (anonymous) institutions. The coordinating center will be able to follow all the participating institutions concomitantly.

For the purposes of this project, we established as a goal a threshold of 85% based on previously reported GWTG results, where clinical outcomes improved when institutions reached this threshold.^28^ Hospitals will be recognized by SBC with a bronze award if they reach this threshold for at least three consecutive months, with a silver award if they sustain these results for at least six months and with a gold award if they continue on the threshold or above it for 12 consecutive months.

## Data analysis

### Data will be analyzed using R program version 3.4.0 or higher.

Hospitals will be excluded from the analysis of a performance measure if less than 10 patients are noted in the denominator for that measure.

Continuous variables with normal distribution will be summarized as mean and standard deviation, and those with skewed distribution as median and 25^th^and 75^th^percentiles. Ordinal or categorical variables will be reported as absolute frequencies, percentages and 95% confidence intervals. Missing data will be addressed on an analysis-specific basis and considered non-compliance for the specific measure.

The longitudinal effect of the program on HF, ACS and AF will be assessed by comparing the overall rates of adherence to the recommendations before and after its implementation in the participating institutions on a quarterly basis, using a generalized linear mixed-effect model (GLMM) for time trend analysis over a time horizon of 18 months. It will be expressed by means of proportions and their respective 95% confidence intervals. It is expected that the random effect approach used by GLMM will account for between-site differences at baseline.^29^

Quality of life scores will be calculated using the methodology reported in the WHOQOL-BREF questionnaire manual.^30^ The total score consists of the average of the scores of the four domains of the instrument (physical health, psychological health, social relationships and environment).^30^ The internal consistency of the instrument will be calculated using the Cronbach’s alpha coefficient. It shall be considered appropriate a value above 0.7.

The results observed over time in the participating institutions on the dependent variables of mortality, readmission rate, length of stay, variation in quality of life and in health perception will be adjusted by multivariable GLMM for demographic, clinical and socioeconomic variables, disease severity, risk factors, initial self-perception of health (NRS), level of health literacy and degree of specific and overall adherence of the institution to clinical recommendations. The variables will be included in the model when associated in the univariate or bivariate analysis (p <0.20) and according to clinical relevance. Odds ratios or relative risks will be calculated, as appropriate, with respective 95% CI.

All analyses will be two-tailed and performed independently for each arm of the protocol using a 0.05 significance level.

## Discussion

### Why is this project needed?

In Brazil, a large country with a complex universal healthcare system,^1^ the quality of cardiovascular care has been the subject of evaluation and concern. Patient access to the various levels of healthcare varies throughout the country and the quality of care delivered is highly heterogeneous.^1 , 2^

As in other parts of the world and in spite of medical society efforts in publishing clinical guidelines, mortality related to CVD remains high, reflecting the difficulty of patients having access to recommended therapies and care at appropriate times.^31 , 32^

Registries performed by SBC in multiple regions of Brazil have shown a high variation in the quality of care delivered for cardiovascular conditions of high economic burden,^32 , 33^ such as coronary artery disease (CAD)^3 , 34^ HF,^4^ stroke, and AF.^35^ These registries have shown that adherence to evidence-based therapies remains suboptimal and, at least for HF, the lack of optimal therapies is more critical in the public non-academic institutions of the poorest regions of Brazil.^4^ It was also observed that morbidity and mortality related to HF are much higher than those observed in developed countries, even when adjusting for region, number of hospital beds and type of institution. The Brazilian registries have contributed enormously in demonstrating how these highly prevalent conditions are being approached across the country, but they have not addressed the gap in the implementation of interventions that may have prevented improvements in the quality of care. Furthermore, they have not controlled for situations where specific therapies are not recommended or are contraindicated.^3 , 4 , 34 , 35^

The two randomized trials (BRIDGE-ACS and IMPACT-AF) performed in Brazil for testing multifaceted interventions to promote adherence to guideline recommendations have shown that the implementation of QI interventions is feasible and can be effective.^6 , 7^ However, these studies did not consider barriers related to local context, did not test if the results observed on adherence to recommendations are sustained over time or the effect of the interventions on patients’ quality of life.^6 , 7^ The BRIDGE-ACS trial, for example, which was performed mostly in academic institutions,^36^ achieved at most 68% adherence to acute therapies and only 51% adherence if all acute and discharge therapies were considered, with no impact on 30-day mortality.^6^ The GWTG program show that hospitals achieving at least 85% of compliance to evidence-based therapies reached better results on clinical outcomes.^37 , 38^

These findings provide a compelling argument in support of the implementation of a QI initiative in Brazilian hospitals that considers the complexity of the local reality and that has already been tested and proven effective elsewhere. The GWTG program, implemented in nearly 50% of all U.S. hospitals, has shown a sustained effect on mortality, length of stay and costs.^39^ There is thus the potential to decrease the economic burden imposed by ACS, HF and AF on the Brazilian health system.

### What is different in the Brazilian program?

Despite the fact that the GTWG program has been deployed in the U.S. for more than 15 years, only as recently as 2016 has another country (China) taken advantage of a similar ACS program.^36^ In Brazil we are starting the program in three different dimensions: ACS, AF and HF. A nationwide quality program focusing on multiple conditions, including outpatient clinics has never been tested within the GWTG experience.^8 , 22^ Also, the notion of patient-reported outcomes including quality of life has been contemplated for the BPC program and may help ministries and cardiology societies in directing health policies to local needs.

The identification of barriers and facilitators in each hospital is considered one of the key steps in the success of clinical implementation strategies. In this project, we are using as a conceptual model a didactic framework proposed by Michie, Stralen and West,^24^ which integrates dynamic and interactive mechanisms to promote behavioral changes resulting from the interaction between the individual (capability and motivation) and the environment (opportunities).^24^ This model will also help the coordinating center in identifying and acting on specific institutional needs during the course of the project. In doing so, in some institutions, intervention will be focused on improving capacity, in others on increasing motivation, and still in others to increase or to restrain the supply of opportunities, individually or jointly, depending on the objectives of each institution. Interventions such as the award program that was considered one of the keys for success in the GWTG experience will be emphasized in all participating institutions.^40^

Lessons learned from the IHI open school experience, such as shaping the audit and feedback intervention with run charts, will be also used in this project.^41^ These approaches consider institutional longitudinal data on the several quality metrics not only in relation to the average benchmarks of the other participating institutions, but also to the goal established for that institution by the median line of the scores obtained for the entire period of observation.^27 , 41^ This feedback loop allows the institution to continuously evaluate itself and redesign processes in rapid improvement cycles,^25 , 26^ considering how their performance differs from the objective and whether adjustments made in their multidisciplinary interventions are resulting in sustained improvement.

## Conclusion

This novel QI program will be provided to selected public institutions in Brazil addressing issues pertaining to the local context that will allow for the identification of specific barriers to the adoption of standards of care. It has the potential to provide solutions that can result in sustained improvement in adherence to evidence-based therapies and patient outcomes.

It is hoped that the implemented strategies will contribute to creating an organizational culture focused on the construction and exchange of knowledge among the institutions nationwide, thereby advancing the quality of cardiovascular health care in Brazil.
